# Glomerular and Mitral-Granule Cell Microcircuits Coordinate Temporal and Spatial Information Processing in the Olfactory Bulb

**DOI:** 10.3389/fncom.2016.00067

**Published:** 2016-07-14

**Authors:** Francesco Cavarretta, Addolorata Marasco, Michael L. Hines, Gordon M. Shepherd, Michele Migliore

**Affiliations:** ^1^Department of Neuroscience, School of Medicine, Yale UniversityNew Haven, CT, USA; ^2^Department of Mathematics “Federigo Enriques”, University of MilanMilan, Italy; ^3^Department of Mathematics and Application “R. Cacciopoli”, University of Naples Federico IINaples, Italy; ^4^Institute of Biophysics, National Research CouncilPalermo, Italy

**Keywords:** olfactory bulb, natural odor, granule, mitral, periglomerular, glomerulus, odor learning

## Abstract

The olfactory bulb processes inputs from olfactory receptor neurons (ORNs) through two levels: the glomerular layer at the site of input, and the granule cell level at the site of output to the olfactory cortex. The sequence of action of these two levels has not yet been examined. We analyze this issue using a novel computational framework that is scaled up, in three-dimensions (3D), with realistic representations of the interactions between layers, activated by simulated natural odors, and constrained by experimental and theoretical analyses. We suggest that the postulated functions of glomerular circuits have as their primary role transforming a complex and disorganized input into a contrast-enhanced and normalized representation, but cannot provide for synchronization of the distributed glomerular outputs. By contrast, at the granule cell layer, the dendrodendritic interactions mediate temporal decorrelation, which we show is dependent on the preceding contrast enhancement by the glomerular layer. The results provide the first insights into the successive operations in the olfactory bulb, and demonstrate the significance of the modular organization around glomeruli. This layered organization is especially important for natural odor inputs, because they activate many overlapping glomeruli.

## Introduction

A network of neurons in the olfactory bulb (OB) implements information processing functions that are necessary for odor recognition. The network is organized into two layers. In the first layer, the olfactory nerves end in modules called glomeruli, where they connect to the dendrites of mitral and tufted cells, and interneurons called juxtaglomerular cells. At the second level, the mitral and tufted cells connect to granule cell interneurons which are modulated by deep short axon cells. The mitral and tufted cells connected to a given glomerulus form what we call a glomerular unit (GU), that is obviously central to processing the olfactory input.

Previous experimental studies by ourselves and others have provided extensive insights into the synaptic organization and functional properties of the granule cells (e.g., Rall et al., 1966; Schoppa et al., 1998; Urban and Sakmann, 2002; Isaacson and Vitten, 2003; Willhite et al., 2006; Bartel et al., 2015), which interact with mitral and tufted cells to carry out lateral inhibition of GUs activated by other glomeruli (Yokoi et al., 1995). The experimental results have been reproduced in detailed realistic 3D models of the mitral and granule cell network (Migliore et al., 2014, 2015).

To understand the full sequence of processing in the olfactory bulb, the olfactory glomerular layer needs to be subjected to the same combination of experimental and computational analysis. Experimental studies indicate that the processing involves complex interactions between the multiple cell types (Aungst et al., 2003; Wachowiak and Shipley, 2006; Whitesell et al., 2013). This presents a much more difficult obstacle to analysis than the relatively direct interactions between the mitral and granule cells at the deep layer. Abstract modeling reflects this complexity (e.g., Benjaminsson et al., 2013), and experimental constraints equivalent to those at the granule cell level are still lacking. As a consequence, there is no realistic 3D model at present for the successive processing that occurs at the two levels in the olfactory bulb.

To begin to address this critical issue, in this work we have built a representation of olfactory glomerular circuits into a three-dimensional (3D) realistic model of the olfactory bulb microcircuits (Migliore et al., 2015). The glomerular circuits incorporate interactions between three main types of cells: mitral cell dendrites, external tufted cells, and juxtaglomerular cells, according to experimental results and theoretical predictions (Cleland and Sethupathy, 2006; Linster and Cleland, 2009). With this as a basis, we have represented these cells and connections as a “glomerular functional unit.” These have been revealed as a spatially distinct cluster or column of cells across the two layers (Willhite et al., 2006). As input, we simulate a set of raw experimental data from natural odor inputs (Vincis et al., 2012). The implementation of glomerular interactions in our 3D model has the effect of decorrelating activity between glomeruli and normalizing olfactory input across different intensities, as suggested theoretically and experimentally (Cleland and Sethupathy, 2006; Linster and Cleland, 2009). We show that this is a fundamental mechanism to ensure an effective action by the next processing stage in the granule cell layer. At this layer the processing involves the temporal decorrelation of the glomerular layer output, with the maximum information transfer taking place within the first 100 ms of the sniff onset.

In summary, the results obtained with this model suggest that a complex input signal is processed by the olfactory bulb in a multistage manner. Each processing layer is independently needed but not sufficient to operate on the input in a specific way in order to obtain an output that will be further decorrelated and recombined over space and time at the next stage, in the olfactory cortex.

## Methods

All simulations were carried out with a fully integrated NEURON+Python parallel environment (NEURON v7.3, Hines and Carnevale, 1997) on a BlueGene/Q IBM supercomputer (CINECA, Bologna, Italy). The model and simulation files specifically used for this work will be available for public download in the ModelDB section of the Senselab database suite (http://senselab.med.yale.edu, acc.n.185318).

Briefly, the model implemented the experimentally-reported spatial distribution of 127 glomeruli distributed in ≈2 mm^2^ of the dorsal area of the mouse olfactory bulb and activated by natural odors (Vincis et al., 2012). The full model was composed of 635 mitral cells (MCs) (5 for each glomerulus) and a total number of 97,017 granule cells (GCs), which uniformly filled the glomerular cell layer of our model (Migliore et al., 2014). This is the total number of GCs and, in principle, all of them would be connected when using a full OB system. In this paper, to take into account the reduced number of mitral and granule cells in our model (about 10% of the real system) the synaptic density on MC lateral dendrites was proportionally reduced to 1 syn/10 μm, instead of the measured average value of 1 syn/μm (Bartel et al., 2015). Also, to take into account experimental indications (Kim et al., 2011), GCs were not allowed to make synaptic contacts with MCs belonging to the same glomerular unit (as schematically shown in Figure S1). The synaptic network configuration was finally built by connecting granule cells to MCs using a collision-detection algorithm and constraints from experimental findings (as explained in Migliore et al., 2014). In the final configuration, each MC was connected with ≈2000 GCs, which received up to 91 synaptic connections from different MCs (within the experimental range; Woolf et al., 1991); the actual number of granule cells that ended up connected to one or more of the 635 MCs was 55,309. The remaining 41,708 were not connected.

The peak excitatory synaptic conductance (from a MC to a GC) was chosen in such a way that during sniffing at the maximum frequency of 10 Hz (Kepecs et al., 2007) the action potentials generated by a MC did not saturate the granule cell spine and, consistent with experimental findings (Labarrera et al., 2013), a granule cell would need a relatively powerful input from mitral cells to elicit a somatic spike. The peak inhibitory conductance (from a GC to a MC, 5.5 nS; from a MC to a GC 1.25 nS) was chosen in such a way to obtain a column approximately 100 μm wide (Migliore et al., 2015), corresponding to the size of a glomerulus and consistent with typical experimental findings (Willhite et al., 2006; Kim et al., 2011). Odor input was implemented as in Migliore et al. (2014), using a peak synaptic conductance, $\bar{g_{\text{max}}}$, scaled to illustrate the specific point of each figure. For the purposes of this work, we used a peak conductance in the range 0–75 nS.

The synaptic plasticity rule was identical to that used in previous work (introduced in Migliore et al., 2007). Briefly, all synaptic weights started at zero and, in response to an odor input, each component (inhibitory or excitatory) of each dendrodendritic synapse changed according to the local spiking activity in the lateral dendrite of the mitral cell or in the granule cell synapse. As discussed in detail elsewhere (e.g., Migliore et al., 2010; Yu et al., 2014), the formation of synaptic clusters consistent with those observed experimentally is a robust process that can be understood by considering the follow dynamics:

1. a strong odor input causes mitral cells to fire at high-frequency;
2. somatic action potentials (APs) backpropagate along the lateral dendrites and potentiate excitatory mitral-granule synapses along their way, activating granule cells;
3. granule cells begin to fire at high-frequency, potentiating their inhibitory synapses on the lateral dendrites of mitral cells.
4. inhibition from granule cells hinders AP back-propagation as it travels far from the soma, thus reducing, locally, the firing frequency of mitral and granule cells, and
5. this finally results in the selective depression of synapses far from the soma of the active mitral cell.

Therefore, as long as: (1) action potentials backpropagate along the mitral cell lateral dendrites, (2) granule cells form dendrodendritic connections, and (3) LTD and LTP are induced by different levels of synaptic activity, a column will form independently from the specific learning rule.

In all cases, simulations were carried out using a 1 Hz sniffing frequency, with each sniff eliciting up to 85 spikes in each mitral cell; stimulation length was 7 s for the learning phase and 5 s for testing.

### Olfactory receptor activity

In particular, the activation of a homogeneous population of ORN is modeled as

$$S_{\text{ORN}}\left(t\right)=O\left(t\right)\left(1-D\left(t\right)\right),\;t\in \left[0,T\right]$$

where *O*(*t*) and *D*(*t*) are the solutions of the following ODEs

$$\{\begin{array}{l} \frac{dO}{dt}\;=\;K_{O}\left(1-C-O\right)\\ \frac{dC}{dt}\;=\;K_{C_{1}}\left(1-C\right)OC+K_{C_{2}}\left(1-C\right),\;t\in [0,t_{1}[\cup ...\cup \left[t_{n},T\right]\\ \frac{dD}{dt}\;=\;K_{D_{1}}\left(1-D\right)O-K_{D_{2}}\left(1-O\right)D \end{array}$$

where *t*_*j*_ is the start time of the *j*-th sniff. In this scheme, there are three states, open (O), closed (C) and desensitized (D). The O and C states reproduce the rise and decay of the signal during a sniff (described in Carey et al., 2009), whereas the D variable implements receptor desensitization, which occurs at high sniffing frequency. Unless otherwise noted, in all simulations we used K_*O*_ = 0.01 ms^−1^, K_*C*_1__ = 0.01 ms^−1^, K_*C*_2__ = 10^−4^ ms^−1^, K_*D*_1__ = 1.7·10^−4^ms^−1^, K_*D*_2__ = 0.01 ms^−1^. The initial condition at the beginning of a simulation (i.e., at *t* = 0) are *O* = 0, *C* = 1 and *D* = 0; at each sniff, C is reset to 0. The overall synaptic current (Destexhe et al., 2001) generated on the mitral cell tuft dendrites by odor activation was calculated as

$$I_{\text{syn}}=g\left(t\right)\left(V_{m}\left(t\right)-E_{\text{exc}}\right),\;t\in \left[0,T\right]$$

where

(1) $$g\left(t\right)=\tilde{g}+\bar{g_{\text{max}}}\cdot \text{GL}\prime \left(c\right)\cdot S_{\text{ORN}}\left(t\right)$$

V_*m*_ is the membrane potential, E_*exc*_ = 0 mV, the $\bar{g_{\text{max}}}$ peak conductance, GL′(*c*) is directly related to the odor identity, concentration, and ORN type (see Equation 4, in the next paragraph) and $\tilde{g}$ implements a random (normal) background activity (0 ± 1 *nS*) taking into account the physiological fluctuations in ORN activation.

### ORN dose-response relations

We started from the relative ORN activation level for 127 glomeruli. We have these data for a set of 19 natural odors at one (suprathreshold, but relatively low) concentration (Vincis et al., 2012).

Experimentally, the ORN activity is represented by dose-response curves, which correspond to the peak I_syn_ current generated for each odor concentration. These curves can be expressed as Hill functions, with different parameters for each odor-glomerulus pair. For example, the overall response of the ORNs converging on glomerulus GL_*i*_ in the presence of odor U at concentration c, can be expressed as (Cruz and Lowe, 2013):

(2) $${\text{GL}}_{i}\left(c\right)=\frac{F_{\text{max}}}{1+\frac{1}{\eta_{i}^{n}}{\left(1+\frac{K_{i}}{c}\right)}^{n}},\;i=0,...,N_{G}$$

where *n* is the Hill coefficient, F_max_ is the maximal response, η_*i*_ is the transduction efficiency for odorant U and N_*G*_ + 1 is the number of glomeruli. The asymptote of each GL_*i*_(c) is

$${\text{asy}}_{i}\equiv \frac{F_{\text{max}}}{1+\frac{1}{\eta_{i}^{n}}}$$

and the concentration of odor U at half maximum response is $\frac{K_{i}}{\sqrt[{n}]{2+\eta_{i}^{n}}-1}$.

In order to compare and analyze the response to different odors we need the odor-response curve for each of our odor-glomerulus pairs. Since this information is not experimentally available, the next step is thus to implement these curves from suitable assumptions for all the parameters. For example, experimentally, the Hill coefficient, n, is quite variable, in the range [0.1,18] (e.g., Rospars et al., 2008; Cruz and Lowe, 2013; Marasco et al., 2016), and F_max_ can be considered to be an intrinsic neuronal property related to the maximum activity that can be generated by any given ORN. For simplicity, in this paper we fixed their value to 2 and 25, respectively, for all cases. From the raw data (Vincis et al., 2012) we have the GL_*i*_ for all of our odor-glomerulus pairs at a single concentration. We call this concentration c_*V*_ and assume c_*V*_=1. For η_*i*_ (modulating the maximal response at high concentrations) and K_*i*_ (related to the concentration at which there is half-maximal activation), there are several experimental findings (e.g., Wachowiak and Cohen, 2001; Rospars et al., 2008; Cruz and Lowe, 2013), showing that each odor-glomerulus pair can exhibit an apparently arbitrary combination of these parameters. To derive their values, it would be necessary and sufficient to find two independent equations for each odor-glomerulus pair. We determined these equations as follows.

Assuming without loss in generality c_*V*_ = 1, for each odor U from Equation (2) we obtain

$$\frac{F_{\text{max}}}{1+{\left(\frac{1+K_{i}}{\eta_{i}}\right)}^{n}}=\rho_{i},\;i=0,...,N_{G}$$

Let us define

$$\rho_{\text{max}}=\underset{{}_{i\;=\;0,...,N_{G}}}{\max}\rho_{i}$$

We assume that in all cases the asymptotic value of the response is proportional to the value at c_*V*_, i.e.,

$${\text{asy}}_{i}=\alpha_{U}\frac{\rho_{i}}{\rho_{\text{max}}}$$

where

$$\alpha_{U}=\underset{i\;=\;0,...,N_{G}}{\max}\frac{F_{\text{max}}}{1+\frac{1}{\eta_{i}^{n}}}$$

is also the asymptotic value of the dose-response curve for the odor-glomerulus pair with the highest input at c_*V*_, i.e., GL_*h*_(c_*V*_) = ρ_*max*_. To determine α_*U*_ we must define a range of possible values of asymptotic response (asy_*i*_) for each glomerular input. From preliminary simulations, we empirically found that the minimum value of asy_max_ to form a column is approximately 1.5 greater than the average value of asy_*i*_. Thus, to ensure that all odors would eventually be able to form a column, if presented at a concentration high enough, we imposed that:

$${\text{asy}}_{\text{max}}-\frac{1}{N_{G}+1}\sum_{i\;=\;0}^{N_{G}}\frac{F_{\text{max}}}{1+\frac{1}{\eta_{i}^{n}}}=\beta$$

With β = 1.5. After standard algebraic manipulations we obtain that

$$\alpha_{U}=\frac{\beta }{1-\frac{1}{N_{G}+1}\sum_{i\;=\;0}^{N_{G}}\frac{\rho_{i}}{\rho_{\text{max}}}}$$

Finally, we can determine the (positive) η_*i*_ and K_*i*_ for all odor-glomerulus pairs by solving the system:

$$\{\begin{array}{l} \frac{F_{\text{max}}}{1+{\left(\frac{1+K_{i}}{\eta_{i}}\right)}^{n}}=\rho_{i}\\ \frac{F_{\text{max}}}{1+\frac{1}{\eta_{i}^{n}}}=\beta \frac{\rho_{i}}{\rho_{\text{max}}-\frac{1}{N_{G}+1}\sum_{i\;=\;0}^{N_{G}}\rho_{i}} \end{array}$$

With β = 1.5, the above system admits positive solutions for all odors. In Table 1 we show ρ_max_ and asy_max_ for all cases.

**Table 1:** **ρ_max_ and α_U_ for all odors**.

**Odor**	**ρmax**	**asymax**
Apple	2.27	8.63
Banana	3.41	4.18
Basil	2.34	9.35
Black Pepper	3.13	5.33
Cheese	2.43	6.52
Chocolate	2.43	10.98
Cinnamon	2.91	5.66
Cloves	2.85	5.76
Coffee	3.04	7.03
Garlic	2.09	12.91
Ginger	2.49	8.67
Kiwi	3.16	5.92
Lemongrass	2.99	6.80
Mint	3.45	5.23
Onion	2.03	17.58
Oregano	2.14	10.51
Pear	2.26	10.90
Pineapple	2.46	9.62
Strawberry	2.28	8.71

### Implementation of glomerular layer circuits

There are different neuron populations at the glomerular layer (GL) level (Aungst et al., 2003). The current view is that they interact among themselves and with an odor input to implement two major mechanisms:

1. an olfactory bulb-wide odor input normalization, and
2. contrast enhancement (CE) generated by a local (intra-glomerular) lateral inhibition (Cleland and Sethupathy, 2006).

There are not enough experimental constraints to implement a biophysically realistic model for each neuron type. The glomerular circuitry in this work was thus represented in terms of a “glomerular functional unit” that carries out input normalization and lateral inhibition between the glomeruli, rather than in terms of explicit cells and synapses. In practice, we closely followed the approach and equations suggested by experimental and computational findings (reviewed in Cleland and Sethupathy, 2006; Linster and Cleland, 2009). A schematic representation of the equivalent microcircuits will be presented in Results.

To take into account the olfactory bulb-wide normalization, we normalized the dose-response curves GL_*i*_ for glomerular input i with respect to the mean over all inputs for a given odor using the transformation:

(3) $$\overset{*}{{\text{GL}}_{i}}\left(c\right)=\{\begin{array}{l} {\text{GL}}_{i}\left(c\right)-\bar{{\text{GL}}_{i}}\left(c\right),\;{\text{if GL}}_{i}\left(c\right)-\bar{{\text{GL}}_{i}}\left(c\right)>0\\ 0,\text{ otherwise} \end{array}$$

where

$$\bar{{\text{GL}}_{i}}\left(c\right)=\frac{1}{N_{G}+1}\sum_{i\;=\;0}^{N_{G}}{\text{GL}}_{i}\left(c\right)$$

Note that, for all c, the $\overset{*}{{\text{GL}}_{i}}\left(c\right)$ values are between 0 and 1.5.

To model the CE effect (Cleland and Sethupathy, 2006; Linster and Cleland, 2009) we assumed that the effective activity of the periglomerular cells projecting to glomerulus *i*, PG_*i*_, can be represented as

$${\text{PG}}_{i}\left(c\right)=\{\begin{array}{l} \frac{a}{1+b\left(\frac{1}{\overset{*}{{\text{GL}}_{i}}\left(c\right)}-1\right)},\text{ if }\overset{*}{{\text{GL}}_{i}}\left(c\right)>0\\ 0,\text{ otherwise} \end{array}$$

where a and b are positive constants. In this case, for all c, the PG_*i*_(c) value will be in the range [0, a/(1-b/3)]. We used *a* = 0.6 and *b* = 0.01.

### Overall input to a glomerulus

The signal on mitral cell tufts was finally calculated as

(4) $$\text{GL}\prime \left(c\right)=\{\begin{array}{l} \overset{*}{{\text{GL}}_{i}}\left(c\right)-{\text{PG}}_{i}\left(c\right),\text{ if }\overset{*}{{\text{GL}}_{i}}\left(c\right)-{\text{PG}}_{i}\left(c\right)>0\\ 0,\text{ otherwise} \end{array}$$

The synaptic conductance in each mitral cell (Equation 1) thus takes into account both the (presynaptic) ORN response to a given odor concentration (Equation 2) and its (postsynaptic) modulation by glomerular processing, Equations (3) and (4). Unless otherwise noted, the overall input in each glomerulus was represented in all figures (using color coded circles) as the average signal activated in the 5 MCs belonging to it.

### Information content carried by synchronous spikes during a sniff

The total simulation time was first divided in bins of equal size, with each bin set to 1 if it contains at least one spike and 0 otherwise. The probability that any two MCs generate a spike within the same time bin, which can be considered as a measure of synchronization, was estimated by exploring the spikes generated within a sliding time window, as explained in detail in Figure S2; the information content carried out by synchronous spikes can be calculated from their probability (see the Results).

## Results

### Odor inputs and olfactory receptor neuron dose-response relations

**Figure 1:**
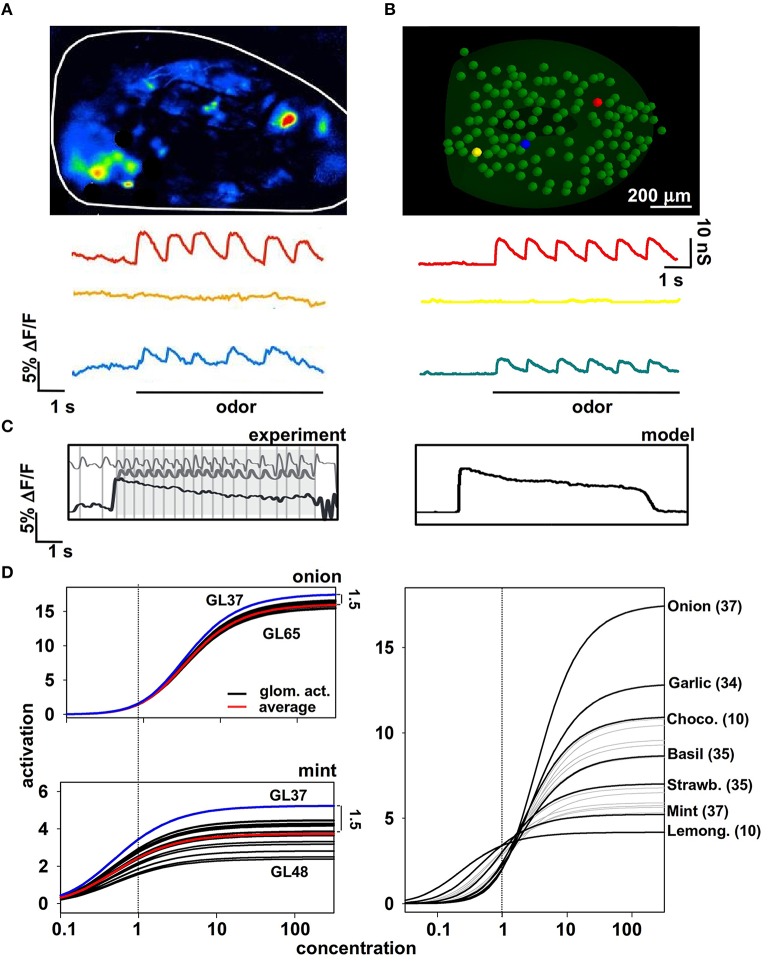
**Modeling odor inputs and olfactory receptor neuron (ORN) activation. (A)** Time course of ORN activation during sniffing activity with a strong (red trace), weak (blue trace), or very weak (yellow) odor input; **(B)** Model results for the synaptic conductance change using the kinetic scheme described in Results for ORN activity; **(C)** experimental findings (left) and model results (right) for ORN activation during sniffing at different frequencies; note signal adaptation at high sniffing frequency (black line). **(D)** The dose-response curves obtained for all glomeruli of all the natural odors for which we have experimental data at a single concentration. See main Methods and text for details. Experimental data in panels **(A,C)** are from Carey et al. (2009).

Information about an odor is contained in the activity of the olfactory receptor neurons (ORNs), which are organized in functional classes, each expressing a particular receptor (Buck, 1993; Sullivan et al., 1994). To understand better the input/output (I/O) operations of the olfactory bulb, it is thus necessary to have first a physiologically plausible representation of the signal that is delivered to any mitral cell, representing an odor and its concentration. This can be expected to be particularly important for natural odors, which appear to activate many ORN types with a complex spatiotemporal distribution (Vincis et al., 2012). We start from the experimental findings (Carey et al., 2009) suggesting that, during a sniff, the axons of a homogeneous population of ORNs converging onto a single glomerulus generate a typical signal with precise temporal pattern dynamics (Figure 1A). These axons release glutamate, which excites AMPA and NMDA receptors on the mitral cell dendritic tufts (reviewed in Shepherd et al., 2004). Importantly, the peak amplitude of this pattern changes with odor identity and concentration, but not its temporal dynamics (Cruz and Lowe, 2013).

To model the experimentally-observed time course of the excitatory signal conveyed to the dendritic tuft of the mitral cells, we used a custom modification (see Methods) of the set of ordinary differential equations based on a generic scheme previously used to model synaptic transmission (Destexhe et al., 1998). The resulting synaptic conductance time course (Equation 1) is shown in Figure 1B for three odor concentrations roughly reproducing the experimentally observations shown in Figure 1A. These equations were also able to reproduce typical ORNs response at high sniffing frequency (Figure 1C). In this way, we have a reasonable representation of the synaptic input in a glomerulus in response to an odor at a specific concentration, but we need to represent the responses to an odor as a function of the concentration, i.e., the dose-response curve. Experimental findings suggest that in almost all cases these curves can be expressed as Hill functions, with different parameters for each odor-glomerulus pair. Following the procedure described in *Methods*, we were able to write a set of Hill functions representing the dose-response curves for a given odor. It is important to stress that, to derive these (experimentally unknown) curves for the response of different glomeruli to the same odor, we used the assumption that the relative ratio of their asymptotic value is the same as that at the reference concentration (*c*_*V*_, see Methods). This constraint is important because it reproduces the progressive recruitment of glomeruli often observed experimentally for increasing odor concentration (Strauch et al., 2012). Two typical examples of odors (“mint” and “onion”) are shown in Figure 1D (left), and the resulting dose-response curve of the most active glomerulus for each odor is shown in Figure 1D (right). It should be noted that concentration in these plots is reported in arbitrary absolute units. Only the relative overall action is important, measured in terms of the peak synaptic conductance that will be activated in the mitral cell tufts to model an odor presentation. Taken together these results suggest one of the possible approaches to extrapolating missing information about odor inputs using the available data and suitable constraints.

### The action of juxtaglomerular circuits on natural odor inputs

**Figure 2:**
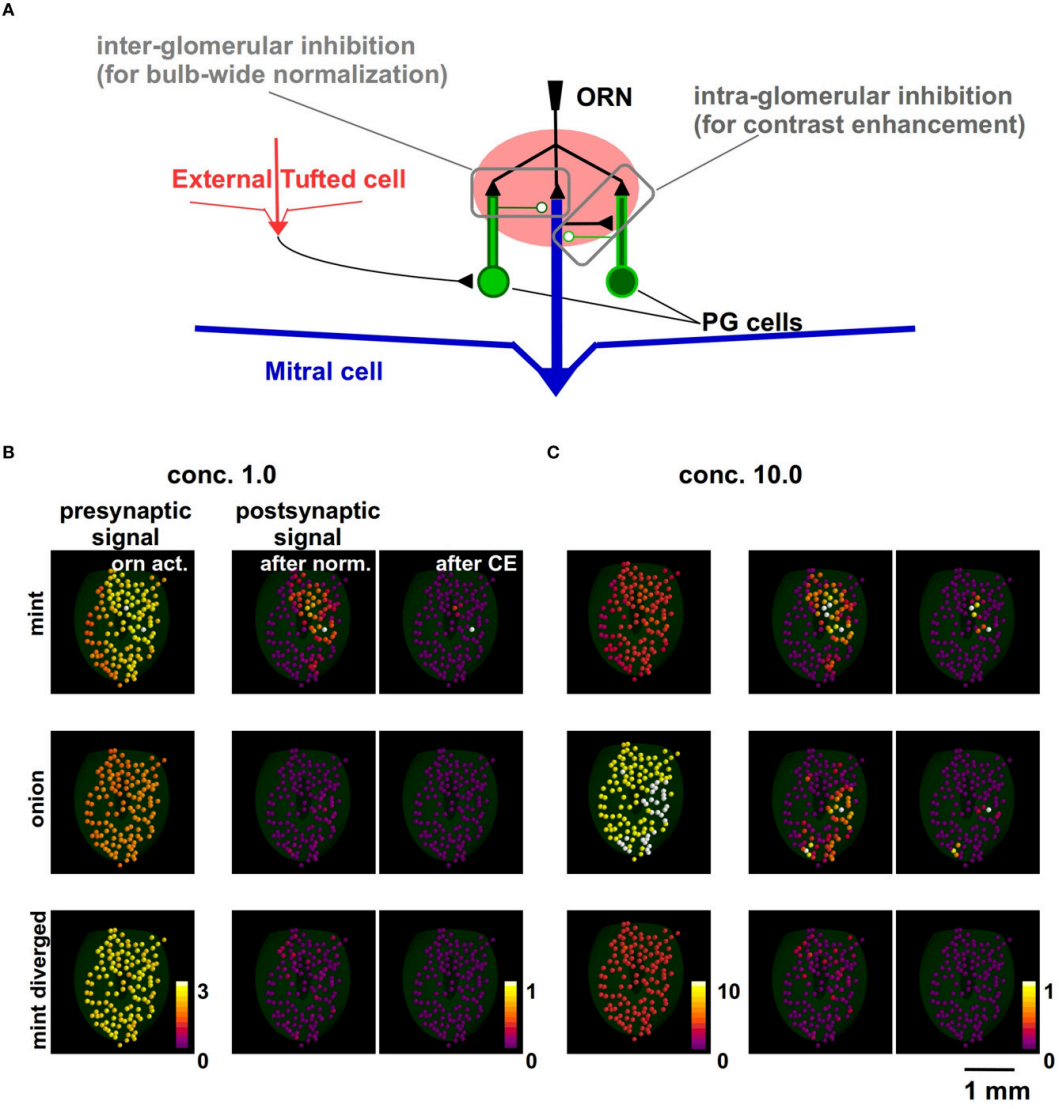
**Modeling how juxtaglomerular circuits transform natural odor inputs. (A)** Schematic representation of the main juxtaglomerular circuits (based on Linster and Cleland, 2009). **(B)** Glomerular activity evoked by ORNs during odor presentation (left), after olfactory bulb-wide normalization (center) and local lateral inhibition, for mint (top), onion (middle), and mint with divergent ORN inputs (bottom). **(C)** Glomerular activation evoked by the same odors presented at higher concentration.

Based on these assumptions about the inputs, we next focused on competing mechanisms acting on the several neuron populations at the GL level (Linster and Cleland, 2009). In Figure 2A we schematically summarize their actions. The olfactory bulb-wide odor input normalization is obtained through the combined action of external tufted cells, short axon cells, and the periglomerular cell (PG) network (implemented by Equation 3). The contrast enhancement (CE) is generated by a local (intra-glomerular) lateral inhibition mediated by periglomerular (PG) cell dendrodendritic interactions (implemented by Equation 4).

To illustrate the consequences of each mechanism on the input, we start with the complex input by the ORN activation levels of 127 glomeruli. We have these data (Vincis et al., 2012) for a set of 19 natural odors at one concentration, c_*V*_. In the left panels of Figure 2B we show the original raw data for mint (top) and onion (middle). For the purpose of this analysis, a third (artificial) odor was built by randomly redistributing the mint inputs over all glomeruli (bottom panel in Figure 2B). It should be noted that, in all panels of Figure 2B, each glomerulus is color-coded using the average normalized synaptic input on the tuft dendrites of the 5 MCs belonging to it.

The implementation of these mechanisms is illustrated in detail in *Methods*. Their overall effect is to implement a kind of winner-take-all effect, shown in the right panels of Figure 2B. The actual value of the peak synaptic conductance was chosen in such a way that the activation of the strongest input in our data (GL37_mint_), during an odor presentation at a relatively high concentration, was able to elicit APs in the mitral cells at a firing rate consistent with experimental observations (up to ≈100 Hz). The same effect is also illustrated in Figure 2C for a higher odor concentration (*c* = 10). Note that this contrast-enhancement effect is at the GL input level, and it will be reflected in the MC output.

Taken together these results support the hypothesis that glomerular layer processing generates a non-topographic contrast enhancement (Cleland and Sethupathy, 2006). The spatial distribution of inputs from natural odors ends up in a configuration of mitral cell inputs in which most of the glomeruli are inhibited below threshold, with a winner-take-all effect that tends to isolate very few strongly active glomeruli. In agreement with experiments (Strauch et al., 2012), stronger inputs (corresponding to higher odor concentrations) will progressively activate additional glomeruli, which will still be bulb-wide normalized by the GL circuit action. Note that, under these conditions, a random divergent input will not activate any glomerulus even at a relatively high concentration.

**Figure 3:**
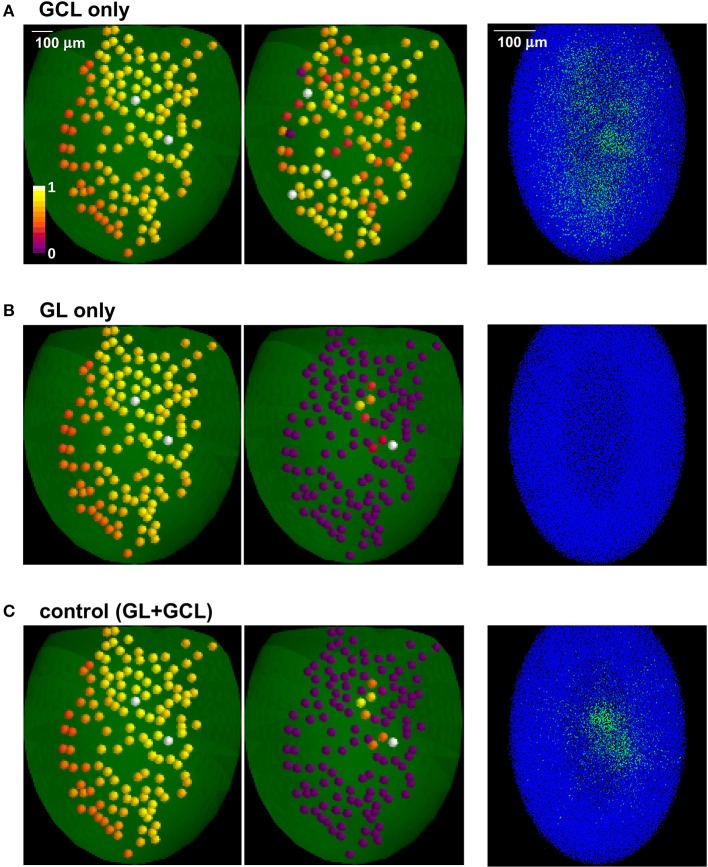
**The role of glomerular and granule cells layers in contrast enhancement**. The glomerular (GL) and granule cell (GCL) layers perform independent processing, in the presence of natural odor inputs; **(A)** with the GCL alone there is no contrast enhancement and odor learning leads to the formation of a diffuse cloud of potentiated GC synapses (right panel); **(B)** GL transforms a dense odor representation (left panel) into a sparse and contrast-enhanced one (center panel) but cannot create columns; both layers **(C)** result in narrow, sparse, and segregated columns (right panel).

In Figure 3 we show the different effects of the two layers. Learning natural odor inputs with the GCL alone (Figure 3A), i.e., without the GL mechanisms, would lead to a diffuse and rather uniform distribution of inhibitory GCs weights with practically no CE effect (Figure 3A, right panel). This occurs because natural odors exhibit a spatially dense glomerular activation (e.g., Vincis et al., 2012). It is easy to see that, in addition to generating a distribution of inhibitory synapses inconsistent with experimental findings (Willhite et al., 2006), this spatially diffuse network configuration would prevent an effective contrast enhancement action on MCs output. The GL alone would transform a dense odor representation into a sparse and contrast-enhanced one (Figure 3B), but cannot generate GCL columns; both layers (Figure 3C) will finally result in narrow, sparse, and segregated columns, in agreement with experimental findings (Willhite et al., 2006; Kim et al., 2011).

## Information content carried by correlated spikes during a sniff

We move next to the second level of this study: how do mitral cells take the input information processed in the glomerular level and encode it for output in the second granule cell level?

It should be stressed that impulse firing of the mitral cell bodies represents the output from the glomerular unit to the olfactory cortex; its firing rate is often used for this purpose (Fantana et al., 2008). However, although firing rates can contain enough information to recognize an odor (Vizcay et al., 2015), during an odor presentation many mitral cells do not exhibit a significant rate change, with respect to the baseline, especially in awake mice (Gschwend et al., 2012). Another way in which information can be encoded is through the spike temporal distribution within a respiratory cycle (Gschwend et al., 2012). One mechanism that can mediate this type of coding is the synchronization of mitral cells from different glomeruli (Giridhar et al., 2011). Experimental findings suggest as noted that the granule-mitral cell inhibition is organized in sparse and segregated columns (Willhite et al., 2006); computational findings suggest that they may form a computational unit with their related glomerulus (Migliore et al., 2015); and in a previous study it was shown how this organization may promote synchronization between mitral cells belonging to different glomeruli (McTavish et al., 2012).

To characterize the information content (Shannon, 1948) carried by the synchronous MC spikes, we first analyzed the inter-glomerular synchronization during a sniff and how it is affected by the mitral-granule cell synaptic network structure.

For this purpose, we needed to evaluate MC spike synchronization from the spike times obtained in any given simulation, and then pool the results for the MCs belonging to a given glomerulus.

**Figure 4:**
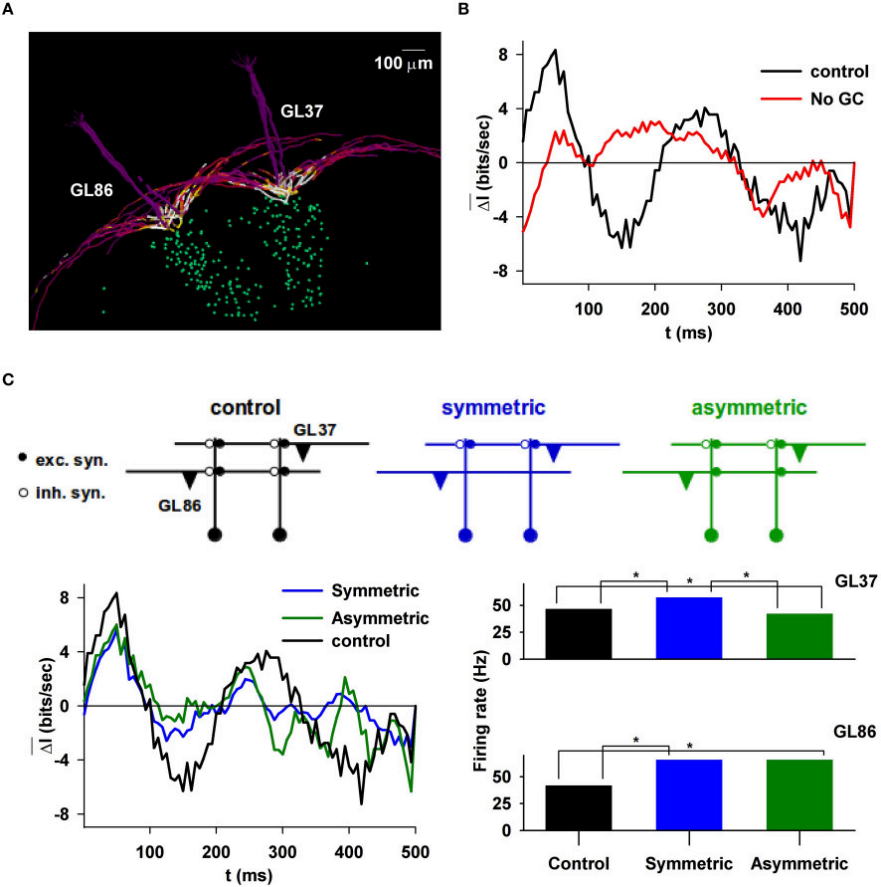
**Information content carried by correlated mitral cell spikes during a sniff. (A)**, Typical example of two glomerular units; **(B)** and the difference in the information content of their spikes during a sniff, with or without granule cells (right); **(C)**, (top) schematic representation of symmetric or asymmetric synapse configuration; (left) information content during a sniff when the column below GL86 was deleted, in the presence of symmetric or asymmetric dendrodendritic synapses; (right) average MC firing rate in the two glomerular units under different conditions, depending on the column present under GL86; marker (^*^) indicates statistical difference.

Thus, we calculated the spike synchronization during a sniff (see Figure S2), and how its information content evolved as a function of time under different conditions. The control condition was a model with two glomeruli approximately 500 μm apart, each one trained with the same stimulus, in such a way to generate a column (Figure 4A). Note that, given the natural physiological variability of mitral cell morphology, included in our model, the columns are not identical. The information content during a sniff was estimated by calculating the average difference between the average information on two MCs firing during a sniff, *log*_2_*(1/p*^*i*^*)*, and its value at time *t, log*_2_*(1/p*${}_{t}^{i}$*)*, i.e.,

(5) $$\bar{\Delta I}=\frac{1}{s}\sum_{i\;=\;1}^{S}\left(\log_{2}\left(1/p^{i}\right)-\log_{2}\left(1/p_{t}^{i}\right)\right)$$

where *s* is the number of sniffs. The average value (from 7 sniffs) under control conditions (i.e., with granule cells) is shown in the right panel of Figure 4B (black line); it was maximal after approximately 50 ms from the sniff onset. Without granule cells (Figure 4B, red line), the information content was significantly lower (Wilcoxon Signed Rank Test, *p*-value < 0.001), suggesting that under this condition no odor information could be propagated to the cortex (Giridhar et al., 2011). Note that negative values of Δ*I* mean that the spikes in the MCs belonging to different glomeruli are less synchronized than average, thus carrying less information. The implication is that the GCL is able to transform the MC output signal in such a way that a relatively large amount of information is transmitted within the first 100 ms from the sniff onset. This range can be related to the overall time course of the inhibitory signal elicited by MCs during their bursting activity. Considering the additional time needed to form a behavioral response, this result is consistent with the experimental findings showing that odor recognition can occur within 200 ms from a sniff onset (Uchida and Mainen, 2003). This time interval must include information passing through other brain regions. Our model suggests that most of the information from the olfactory bulb is transmitted within the first 100 ms (with a peak at about 50 ms).

Odor input, in principle, can stimulate any glomerulus. This will occur independently from the presence of a column. The lack of a well-formed column in general may result in reciprocal synapses that lack one or both of the excitatory or inhibitory components. These configurations, termed symmetric or asymmetric, were predicted by our model (Migliore et al., 2010) and recently observed experimentally (Bartel et al., 2015). They are schematically illustrated in the top plot of Figure 4C for the two-glomeruli model used in this case. To test their effect, we calculated the difference in information content from simulations in which only one column was present, below GL37. With respect to control, the information content is significantly lower when there is only one column (Figure 4C, bottom left, compare black with blue/green curves), independently of the presence of symmetric (*p* < 0.001) or asymmetric (*p* = 0.011) synapses on glomerulus 86 (GL86). However, asymmetric synapses can induce lateral inhibition and can affect the spike train in a significant way. As shown in Figure 4C (bottom right), in the presence of asymmetric synapses (green bars) GL86 decreased significantly the GL37 firing rate, whereas the opposite (GL37-mediated inhibition on GL86) is not possible (Figure 4C, bottom right, compare green and blue bars for GL86). This effect may have functional consequences for odor discrimination, because it would reduce the interaction between glomeruli activated by a relatively “new” odor (i.e., for example with an asymmetric column) and other glomeruli previously involved with other odors (i.e., with a well-formed, and symmetric, column).

In summary, lateral inhibition through GCL circuits is a basic mechanism for implementing interglomerular communication and shaping synchronous spike distribution across the sniff time course, maximizing the information content carried by spikes. We hypothesize that the GL circuits are not involved in this effect, because it requires a reciprocal lateral inhibitory mechanism. Such a mechanism cannot rely on the GL circuit, which implements a feedforward inhibition. The role of the GL circuit in this process cannot be studied in more detail in this work, where we implemented glomerular microcircuits with an effective set of (experimentally constrained) equations rather than with explicitly interacting cells. When more experimental constraints on morphology, electrophysiology, connectivity, and synaptic plasticity of GL circuits become available, they can be readily introduced into the model and test additional hypotheses.

### Olfactory bulb network configuration as a function of learning

In the previous sections we have studied the interactions between isolated glomerular units. Complex odors however require interactions among many units. We have therefore used our full model, which has led to new insights into mechanisms related to learning of natural odors.

We first note that, since a column can form only below the glomeruli which are strongly active during odor training (Migliore et al., 2007), the presence of a column in a particular spatial location in the olfactory bulb can be related to odor identity and concentration. Furthermore, a column can also affect information propagation and decorrelation of other columns (Migliore et al., 2015). Experimentally, the most important mechanism from this point of view seems to be the decorrelation that an odor pattern undergoes after a few hundred milliseconds from a sniff onset (Niessing and Friedrich, 2010). It is therefore important to explore this issue in more detail, starting from the process of column formation following training with different odors in the full model.

**Figure 5:**
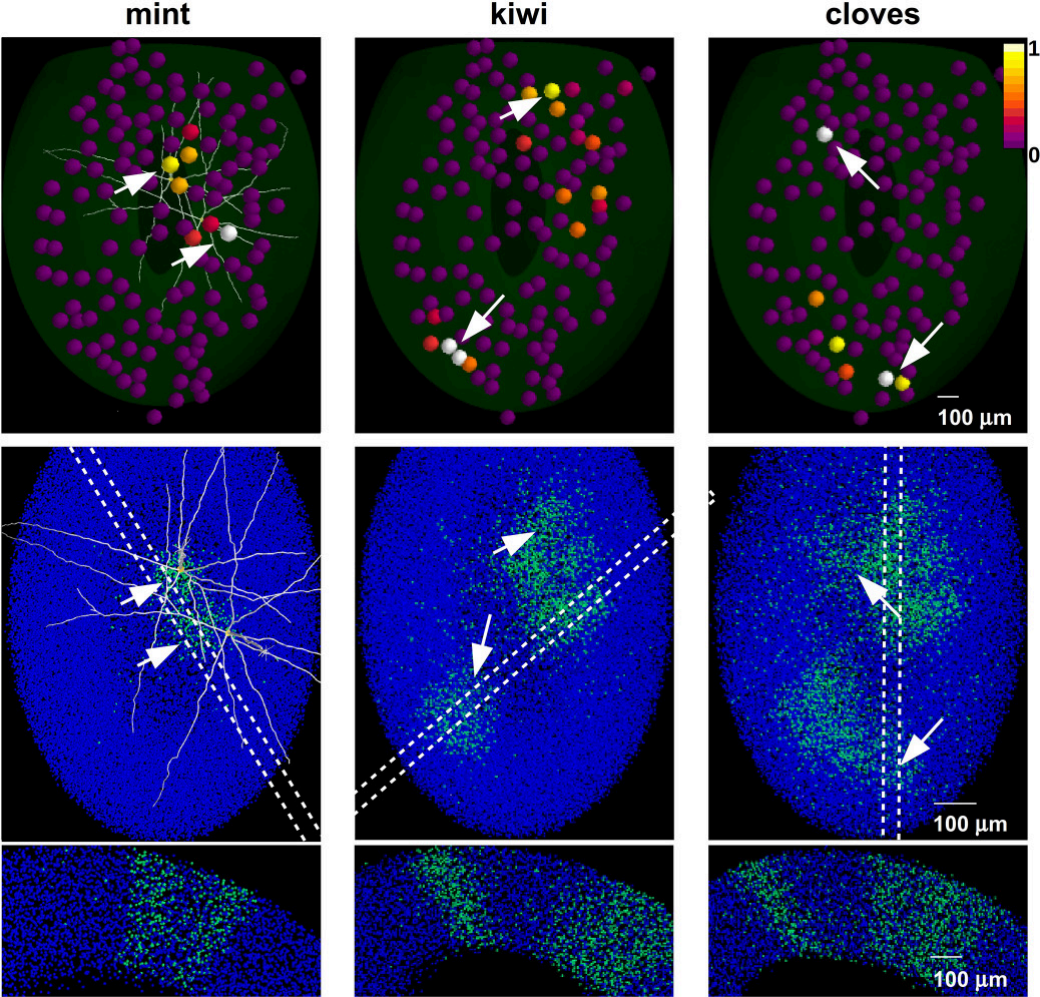
**Mitral-granule cell network configuration after learning different odors**. **(top)** Glomerular activity evoked by presentation of mint **(left)**, kiwi **(center)** and cloves **(right)**. New columns are formed after each odor presentation **(middle)**. Arrows mark the most active glomeruli and related columns. The dashed regions indicate possible slices, equivalent to those carried out experimentally, in which columns can be better observed **(bottom)**.

For each natural odor we fixed a concentration level (Table 2) to have at least one glomerular unit sufficiently activated to form a column. Odors mint, kiwi, and cloves were thus sequentially presented as inputs. In all simulations, every odor lasted for 7 and 5 s during the learning or testing phase, respectively, whereas all glomeruli were activated every second to reproduce a sniffing frequency of 1 Hz. The column configuration after each odor presentation is illustrated in Figure 5, where we plot the spatial distribution of the inputs on the MC tufts (Figure 5, top), the network configuration in terms of granule cells with strong synapses (Figure 5, middle), and the column distribution that would be observed with different slices (Figure 5, bottom). It is interesting to note how the shape, size, and distribution of the columns reproduce the same features observed in experiments (Willhite et al., 2006; Kim et al., 2011); new, relatively well-demarcated, columns were formed after every odor learned.

**Table 2:** **Odor concentration used for all simulations with GL**.

**Odor**	**Concentration**
Cloves	2.07
Banana	0.55
Lemongrass	2.31
Mint	2.36
Basil	3.8
Pineapple	4.67
Oregano	4.72
Cinnamon	4.66
Garlic	6.95
Strawberry	10.0
Chocolate	8.86
Ginger	4.83
Black Pepper	1.83
Kiwi	6.95
Pear	6.16
Onion	5.46
Apple	8.86
Coffee	2.42
Cheese	4.28

**Figure 6:**
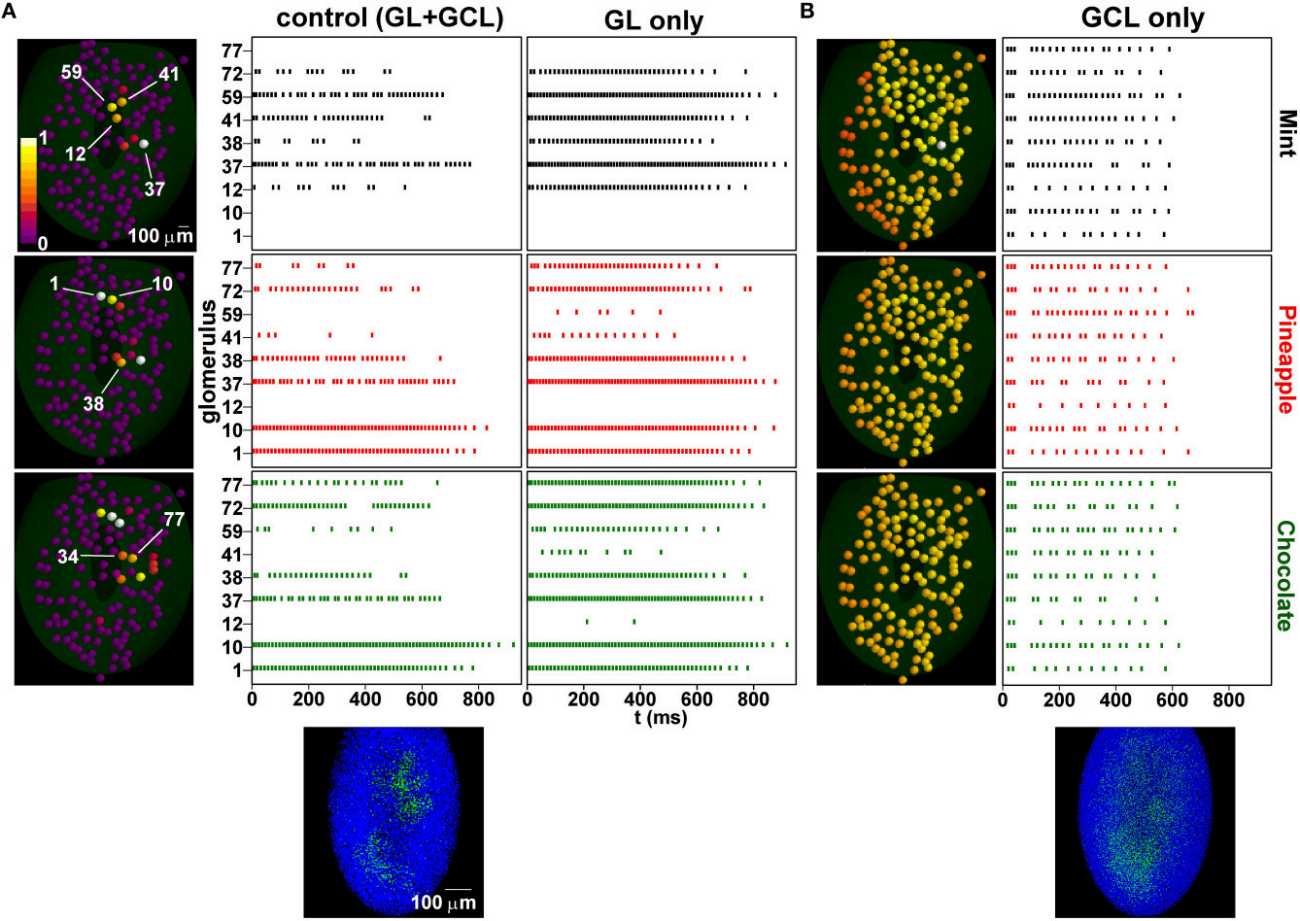
**Learning increases spatial decorrelation of inputs. (A)** (left) Glomerular unit activity evoked by presentation of mint (top), pineapple (center), chocolate (bottom); (middle) Raster plots of mitral cells belonging to different glomeruli under control, after training with the sequence mint-kiwi-cloves; the bottom plot shows the configuration of inhibitory synapses of granule cells after learning; (right) without the granule cell layer; **(B)** same as in **(A)** but after learning and without the glomerular layer.

To see how this network configuration affects unknown odor inputs, we studied, with and without the GL, the MC firing pattern evoked by three odors: mint (known), pineapple (unknown), and chocolate (unknown). The results are shown in Figure 6. Input and average activity after single odor learning are shown in Figure S3. We chose these odors because of their somewhat overlapping glomerular activation, schematically represented in the left panels of Figure 6A. Training with both layers (Figure 6A, center panels), resulted in most glomeruli responding with different patterns for different odors, and well-defined columns (Figure 6A, plot below control panels). This effect was clearly correlated with both lateral and feedback inhibition. Although MCs not involved in the training period (e.g., 1 and 10, activated by pineapple and chocolate) did not show any significant change, as they did not have any associated column, most of the other MCs showed some sign of interaction, in the form of change in the spiking temporal distribution. From this point of view, an entire repertoire of features can be distinguished already with this relatively simple configuration, from complete inhibition of glomerular unit activity (e.g., 12, 59, 41) to characteristic bursting properties (e.g., 12, 38, and 72) that depend on the specific spatial interaction among active glomerular units and could be used to identify an odor input. Overall, these patterns were similar to what has been observed experimentally (Shusterman et al., 2011). Moreover, those mitral cells associated with a column exhibited a burst-like activity during the ORN stimulus time course, due to the feedback inhibition evoked from the connected GCs. This is consistent with experimental results, which show a burst-like activity when a current was injected during the simulated ORN response pattern (Chen and Shepherd, 1997). Without the GCL (Figure 6A, right panels), all odors evoked a rather strong activity. Many glomerular units were more or less activated by all three odors, with spiking patterns that lasted for the entire time course of the mitral cell tuft response to ORN activation (see Figures 1A,B). Without the GL processing, odor presentation evoked a dense glomerular activity (Figure 6B, left panels). Under this condition, training formed a diffuse cloud of inhibitory GCs weights (Figure 6B, bottom left) that resulted in all glomerular units substantially responding to all odors in a similar way (Figure 6B, right panels). Taken together, these results demonstrate why both layers are needed to process natural odors.

### After learning, the spatial overlap in average firing rate decreases over time

**Figure 7:**
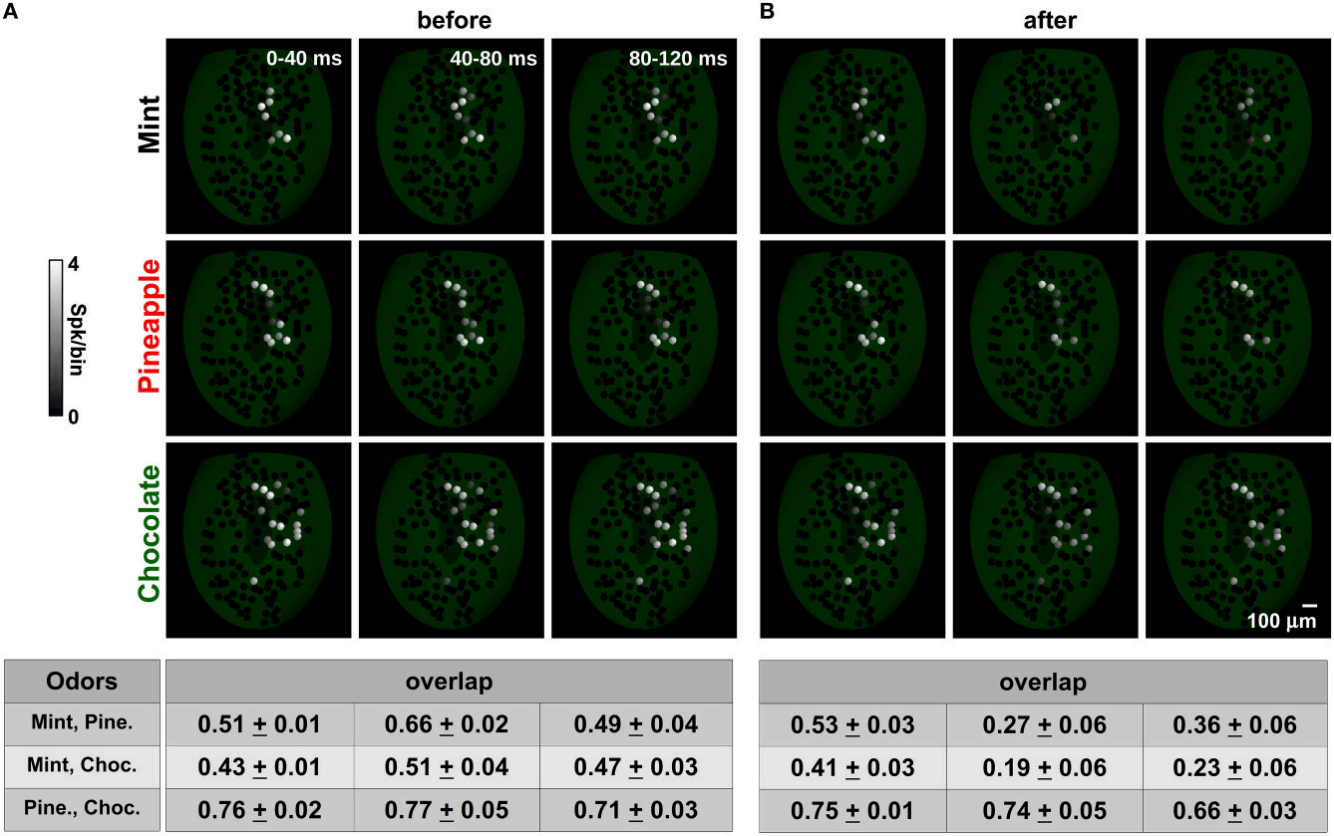
**Spatial mitral cell activity decorrelates over time after learning. (A)** The average spatial activity for mint (top), pineapple (medium) and chocolate (bottom) at different time bins during a sniff before learning. A spatial overlap for each pair is shown in the table at the bottom for each time window; **(B)** same as in **(A)** but after learning mint, kiwi, and cloves.

For a more quantitative measure of the effects on glomerular unit interactions during a sniff, we analyzed how the spatial activity patterns were affected by training at different time windows from the sniff onset (0–40, 40–80, and 80–120 ms). For this purpose, we calculated the spatiotemporal overlap, between any given odor pair (Figure 7, bottom), as the cosine between the vectors formed by the average spike number of the mitral cells belonging to each glomerulus within each time window. Because glomeruli have a fixed spatial location, these vectors can represent the spatial activity pattern of the olfactory bulb. Each pair exhibited a distinct level of spatial overlap, depending on their spatial and temporal activation.

Before training (Figure 7A), odors exhibited more or less overlap that depended on the spatial distribution of the active glomeruli. Chocolate was relatively less overlapping with mint with respect to pineapple during the entire observed period (compare overlap table for mint-choc and pine-choc in Figure 7A). Mint and pineapple instead exhibited a time dependent overlap that was maximum in the 40–80 ms window. These values were entirely dependent on the relative strength and time course of the glomerular input for each odor. It should be noted that this configuration corresponds to an OB in which there are only GL circuits.

Training (Figure 7B) resulted in an overall activity reduction in nearly all glomeruli, especially those activated by an odor already known to the network (such as mint), and thus with well-formed columns (see the right panel of Figure 3C and the left panels of Figure 5). Although this reduction was already evident in the time window just after the stimulus onset, the overlap among odor pairs (especially for those unknown to the network) was not affected during the same period. However, it was systematically reduced in all odor pairs during the time course of the stimulus. It should be stressed that this effect cannot be obtained without the GL circuit (Figure S4). These results suggest that the granule cells can strongly change the spatiotemporal structure of the mitral cell spikes in such a way as to disambiguate similar input patterns. The overall effect is consistent with experimental findings (Abraham et al., 2004; Niessing and Friedrich, 2010), and our model predicts that it will be stronger for odor pairs containing a known component (mint, in this case) and lower for unknown odors.

In summary, odor learning reduced the relative overlap between each odor couple, therefore enhancing the differences between the related spatial activity patterns and the odor discrimination abilities of the olfactory bulb. This is in accord with cognitive testing that has revealed that odor learning gives significantly improved discrimination accuracy in rats (Fletcher and Wilson, 2002; Rokni et al., 2014; Gschwend et al., 2015).

The overlap reduction is dependent on the presence of well-formed columns, which are essential for this mechanism to work. Columns with an inhibitory action that is not spatially segregated or strong enough will not work well to reduce the input overlap. A column's size, shape, and overall effect on action potential backpropagation depend on the peak inhibitory synaptic conductance (Migliore et al., 2015) and, more generally, on the balance between excitation and inhibition (Yu et al., 2014). The control conditions used in our model can be considered as balanced, from this point of view.

We then tested how a column can change the input overlap using peak inhibitory conductance values half or double compared with control. After training, the same odors were presented to compare the relative overlap average between each odor couple. The results reported in Table 3 show how significant deviation from a balanced (control) condition results in a worse and less stable reduction of the overlap between input patterns, suggesting an impaired discrimination of odors.

**Table 3:** **Overlap in average firing rate for different peak inhibitory conductances**.

	**0–40 (ms)**	**40–80 (ms)**	**80–120 (ms)**
Control	0.57 ± 0.15	0.40 ± 0.25	0.42 ± 0.19
reduced inh. (half)	0.57 ± 0.15	0.47 ± 0.21	0.44 ± 0.18
increased inh. (double)	0.56 ± 0.14	0.37 ± 0.30	0.46 ± 0.18

**Figure 8:**
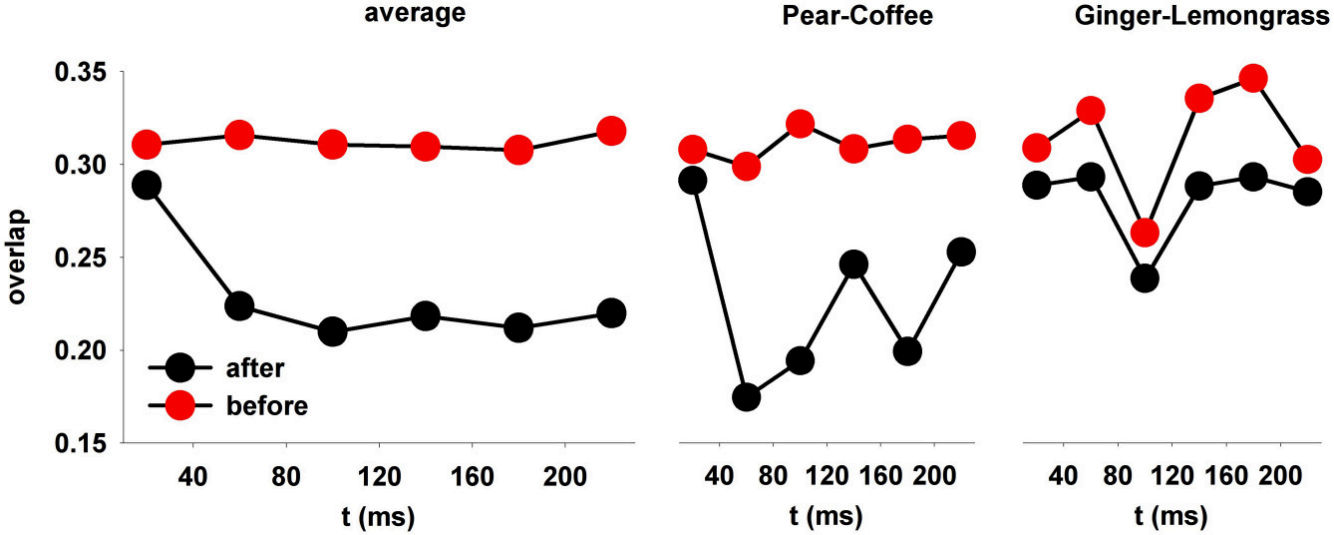
**After learning, the spatial overlap in average firing rate decreases over time**. **(left)** The overlap in MC firing rate during a sniff, averaged over all odor pairs; **(right)** the relative overlap of two specific pairs, pear-coffee, and ginger-lemongrass.

The average change in the overlap, obtained after testing the model trained with 3 odors, was calculated from all possible pairs of 19 natural odors (Figure 8). Before training (Figure 8, red symbols), the overlap was relatively high and constant throughout the sniff. After training, it was significantly reduced within 80 ms (*p* < 0.001) within all time windows for each odor couple. This was observed for many (but not all) pairs, as shown in the middle and right panels of Figure 8.

In summary, these results show what computations are performed by the granule cells during odor learning. Granule cells decorrelate the odor representation by glomerular units over time. For this to occur, well-formed columns are necessary. These results help to explain why the olfactory cortical representation of odors exhibits a reduced overlap compared to the glomerular layer (Stettler and Axel, 2009), and why zero-noise correlation occurs between the neurons of the anterior pyriform cortex during odor recognition (Miura et al., 2012).

### Odor learning affects the relative spatial overlap between odor pairs

**Figure 9:**
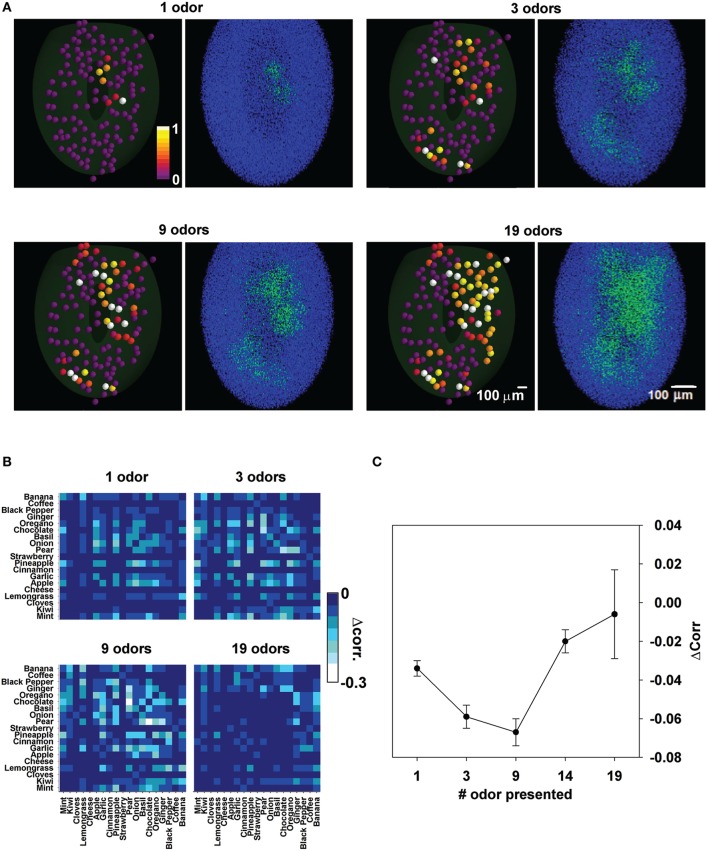
**Odor learning affects the relative spatial overlap between odor pairs**. **(A)**, Input and network configuration after learning of 1, 3, 9, and 19 odors; for each case we plot the maximum input to each glomerulus (left panels) and the corresponding final configuration of inhibitory synapses (right panels). **(B)** Decorrelation matrix for the spatial overlap of average firing rates for different number of learned odors. **(C)** Average decorrelation as a function of odor learned.

We finally consider that with learning of an increasing number of odors, it may be predicted that the columns will gradually merge into a large, structurally undefined, set of strongly potentiated synapses (Figure 9A). This could be especially true for complex natural odors, as those explored in this work.

To study this effect, the difference in the correlation between odor pairs before and after training with different odors was calculated (Figure 9B). Brighter pixels indicate a progressively larger decorrelation. As can be gathered by the increasing number of brighter pixels, odors were more and more decorrelated with training. However, after training with all 19 odors the decorrelation appeared to be much reduced or absent. In Figure 9C we plot the average change in correlation between any two odors as a function of the number of trained odors. Taken together these results suggest that there may be an optimal number of odors on which the olfactory bulb can operate at any given time.

## Discussion

The overall picture emerging from the results of this study is one in which a complex odor signal is processed in a multistage manner, at the glomerular and granule cell layers (GL and GCL, respectively). Each processing layer is independently needed (but not sufficient) to operate on the input in a specific way. We summarize the findings and their interrelation with regard to contrast enhancement in the glomerular layer and temporal decorrelation in the granule cell layer.

**Figure 10:**
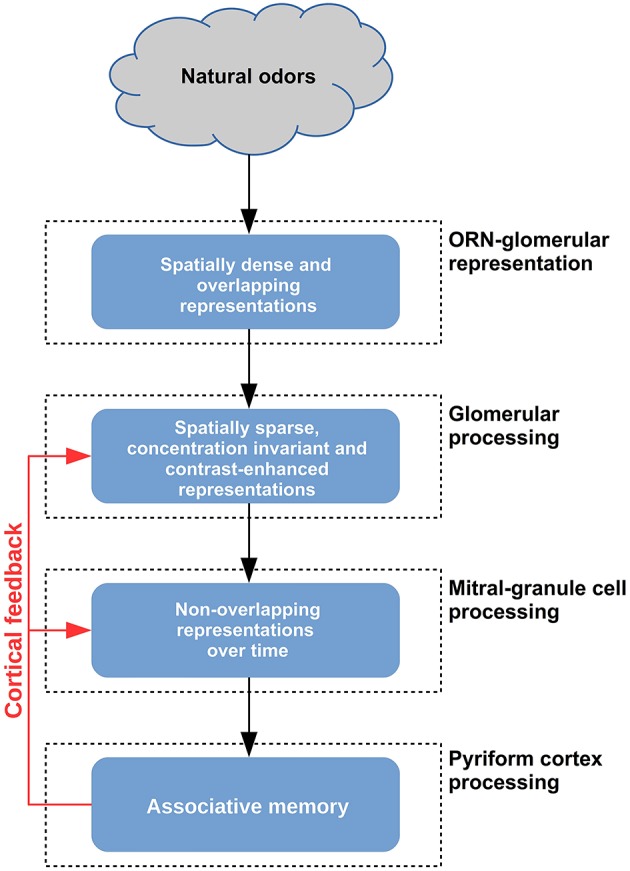
**Schematic representation of the functional consequences of glomerular and granule cell layers in the olfactory bulb**.

To begin, an initial preparatory stage takes place in the olfactory neuron input. As summarized in Figure 10, natural odor molecules have a dense input representation in terms of populations of activated glomeruli (Vincis et al., 2012), even at relatively low concentrations. In contrast, monomolecular odor molecules activate only few glomeruli. Representing this natural odor distribution is novel in our 3D model (Migliore et al., 2014). We show here that it is key to the sequence of operations.

At the next stage (Figure 10, second layer), the fundamental role of the GL is to make the input spatially decorrelated and sparse. This is accomplished through a winner-take-all processing that selects only strongly active glomeruli (Figure 2). We have shown that this process cannot be implemented by the GCL in the presence of the dense input activated by natural odors (Figures 3, 5B). The effect of this mechanism, mainly operating through juxtaglomerular cells, was shown in a reduced network of the glomerular layer with simplified artificial inputs (Linster and Cleland, 2010). Here we extended its validity and scope by applying it in our realistic 3D model.

At the final stage (Figure 10) in the granule cell layer we show that several critical operations take place. GCL processing is needed for column formation and interaction, during learning, and to add temporal processing and additional spatial decorrelation, during odor presentation. These mechanisms operate following the odor-dependent activation of the mitral cells and their reciprocal synapses within the granule cell network; according to the columns present at any given time, the mitral cell output in the presence of different inputs is spatially sparse and decorrelated over time (Figure 7B). We show that the process cannot be properly implemented without a GL preprocessing (Figure S4).

The model suggests that this interaction can be especially important during odor learning, which relies on synaptic plasticity at the mitral-granule circuit level. We stress that, although synaptic plasticity has not been directly observed in the reciprocal mitral-granule cell synapses, there are several indirect experimental findings suggesting its occurrence in the olfactory bulb (Mandairon and Linster, 2009) and in the mitral (Ennis et al., 1998; Ma et al., 2012) and granule cells (Gao and Strowbridge, 2009; Arenkiel et al., 2011). This is an important issue, and we plan to investigate alternative hypotheses in a future work.

Our approach makes it possible to put into this same framework a number of theoretical and experimental findings. Juxtaglomerular cells in the GL act through interglomerular and local interaction with the mitral cells (Cleland and Sethupathy, 2006; Linster and Cleland, 2009). By contrast, granule cells in the GCL operate on mitral cells through feedback and lateral inhibition over time and a larger spatial domain. This temporal processing and synchronization was predicted in the original description of the dendrodendritic interactions (Rall et al., 1966; Rall and Shepherd, 1968). Their concurrent action is such that, within ≈150 ms from the stimulus onset (Uchida and Mainen, 2003), the output of the olfactory bulb is temporally morphed (Niessing and Friedrich, 2010) and spatially organized to form a code that is theoretically sufficient to explain all of the human and rodent abilities to discriminate odors (Koulakov et al., 2007).

The model results especially highlight the fundamental role of GL circuits for processing natural odors. Under the evoked dense spatial activation of glomeruli (Vincis et al., 2012), the granule cells cannot form the narrow and well-defined columns that are needed to decrease the relative overlap between odor representations after learning. The overall picture is consistent with experimental findings suggesting that the lateral inhibition relies more on the glomerular layer circuits, whereas the relatively lower granule cell inhibition is likely to implement mitral cell synchronization (Whitesell et al., 2013).

An interesting prediction of the model is the limitation on the number of odors that can be learned by the olfactory bulb before reaching its computational limit. Although we did not test low odor concentrations, which can result in column erasure (Migliore et al., 2010), it should be clear that presentation of a number of odors at concentrations high enough to form a column will eventually overwhelm the sparse and distributed glomerular column organization. We hypothesize that external mechanisms, such as neurogenesis, cortical feedback (Otazu et al., 2015), or neuromodulatory inputs from other brain regions, may help to expand this limit. These results have all been obtained with a model based on the glomeruli in the dorsal region representing approximately 10% of the olfactory bulb. Obviously the rest of the olfactory bulb greatly expands the numbers of odors that can be discriminated.

Finally, the model results in decorrelation of odor pairs, suggesting the behavioral prediction that specific odors pairs may be discriminated according to individual recent odor experience. The direct use of experimental data on natural odors allows specific predictions, assuming that the distribution of the inputs in the dorsal part of the olfactory bulb is a good representation of the odor. Thus, for example, in the model, recent odor learning of mint, kiwi, and cloves should result in a better discrimination between pear and coffee, and a much more confused discrimination between ginger and lemongrass. The next step will be to extend this approach to the entire olfactory bulb, where it is known from other activity marking methods such as 2-deoxyglucose (Stewart et al., 1979; Johnson and Leon, 2007) and functional MRI (Xu et al., 2003) that most odor activation with monomolecular odors occurs. It will therefore be critical to test activation of the entire olfactory bulb with natural odor stimuli.

## Author contributions

FC, GS, and MM designed research; FC performed research; FC, MH, and AM contributed new analytic and simulation tools; FC and MM analyzed data; AM, GS, and MM wrote the paper.

### Conflict of interest statement

The authors declare that the research was conducted in the absence of any commercial or financial relationships that could be construed as a potential conflict of interest.
