# Supplementary material for: Glomerular and Mitral-Granule Cell Microcircuits Coordinate Temporal and Spatial Information Processing in the Olfactory Bulb
Source: Front Comput Neurosci. 2016 Jul 14;10:67. doi: 10.3389/fncom.2016.00067 (PMC4943958; doi:10.3389/fncom.2016.00067)
Supplement: Supplementary file 1 [file Image1.PDF]

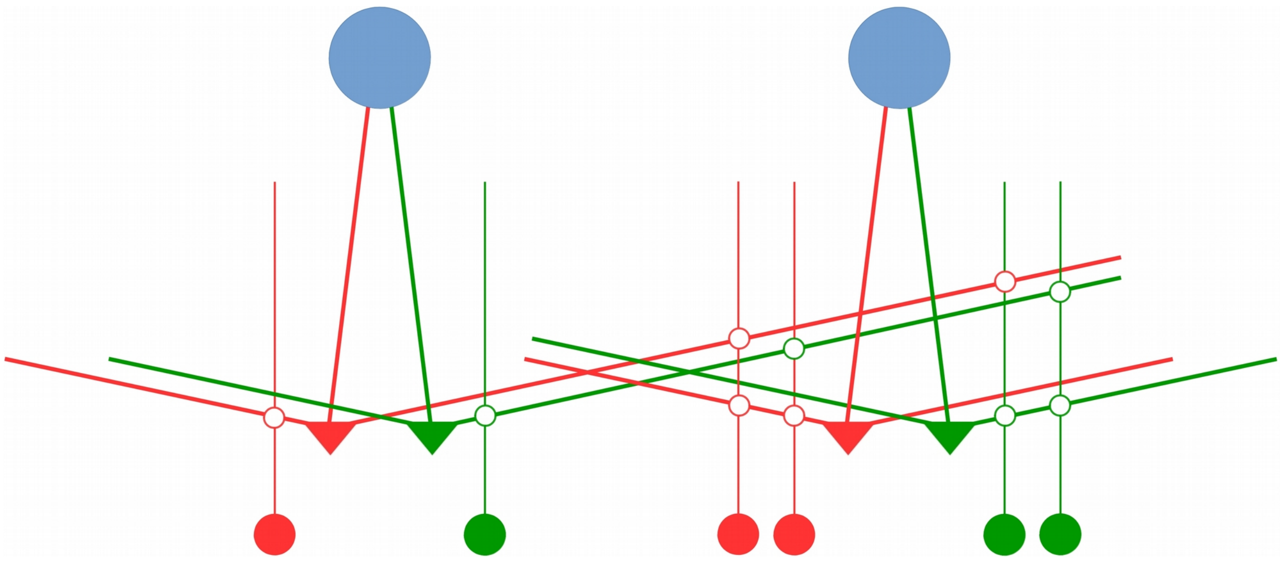

Figure S1. Intra- and inter-glomerular unit connectivity.
A granule cell is limited in its own synaptic connections to its own mitral cell within a glomerular unit (left), but can connect to dendrites from any mitral cell belonging to other glomerular units (right). Based on data from Kim et al., 2011.
