# Supplementary material for: Glomerular and Mitral-Granule Cell Microcircuits Coordinate Temporal and Spatial Information Processing in the Olfactory Bulb
Source: Front Comput Neurosci. 2016 Jul 14;10:67. doi: 10.3389/fncom.2016.00067 (PMC4943958; doi:10.3389/fncom.2016.00067)
Supplement: Supplementary file 2 [file Image2.pdf]

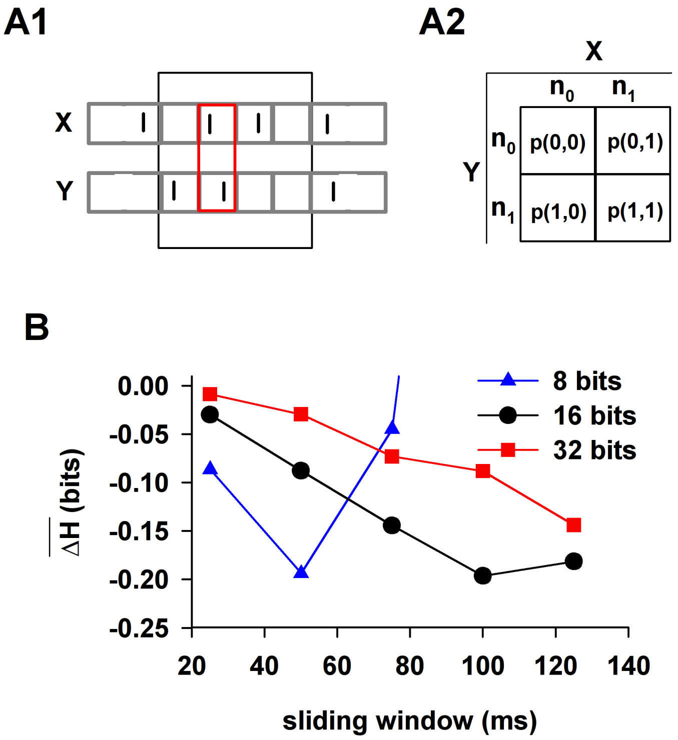

Figure S2. Spikes train analysis used to calculate the information content of synchronized spikes. The total simulation time was first divided in bins of equal size, with each bin set to 1 if it contains at least one spike and 0 otherwise, as schematically represented in A1. Two vectors were formed with two spike trains from any two given mitral cells connected to distinct glomeruli. A contingency table was then calculated based on the results obtained by exploring the two vectors with a sliding time window, as schematically shown in A2. A fundamental step is the choice for the sliding window and the bin size, which must be chosen in such a way to capture the maximum amount of information on MC synchronization. This can be done by considering that without granule cells the activity in any two glomerular units will have a higher average joint entropy, because the MC spikes are not expected to be correlated. In light of the principle of maximum entropy (Jaynes ET, 1963), the best combination of time window and bin size is thus that resulting in the maximum reduction of the joint entropy between spike trains obtained with or without granule cells; in such manner it is discovered the combination which intercepts the maximum information carried from the mitral cells spikes about the stimulus.In general, the joint entropy, i.e. the entropy of a joint probability distribution (represented in our case by the contingency table) is calculated as

$$H\left(X_t, Y_t\right) = \sum\nolimits_{a,b\in\{0,1\}} -p\left(a, b\right) log_2 p\left(a, b\right)$$

where X_t and Y_t are the discretized spikes inside a time window centered at t, and *p(a, b)* is the probability to have a given value for *a* and *b* in the contingency table for X_t and Y_t.

The probability that any two MCs generate a spike within the same time bin can be considered as a measure of synchronization. To capture the amount of information from the simulations, we tested sliding windows of 25-125 ms partitioned in 8, 16, or 32 bins, to understand which combination results in the maximum average difference in the joint entropy between simulations with and without granule cells. It was found (B) that a 100 ms (16 bin) sliding time window gave the best result.

References

Jaynes, E.T. (1963). Information Theory and Statistical Mechanics, in Statistical Physics, ed. Ford, K. (New York: Benjamin), p. 181.
