# Supplementary figures and images for: Glomerular and Mitral-Granule Cell Microcircuits Coordinate Temporal and Spatial Information Processing in the Olfactory Bulb
Source: Front Comput Neurosci. 2016 Jul 14;10:67. doi: 10.3389/fncom.2016.00067 (PMC4943958; doi:10.3389/fncom.2016.00067)

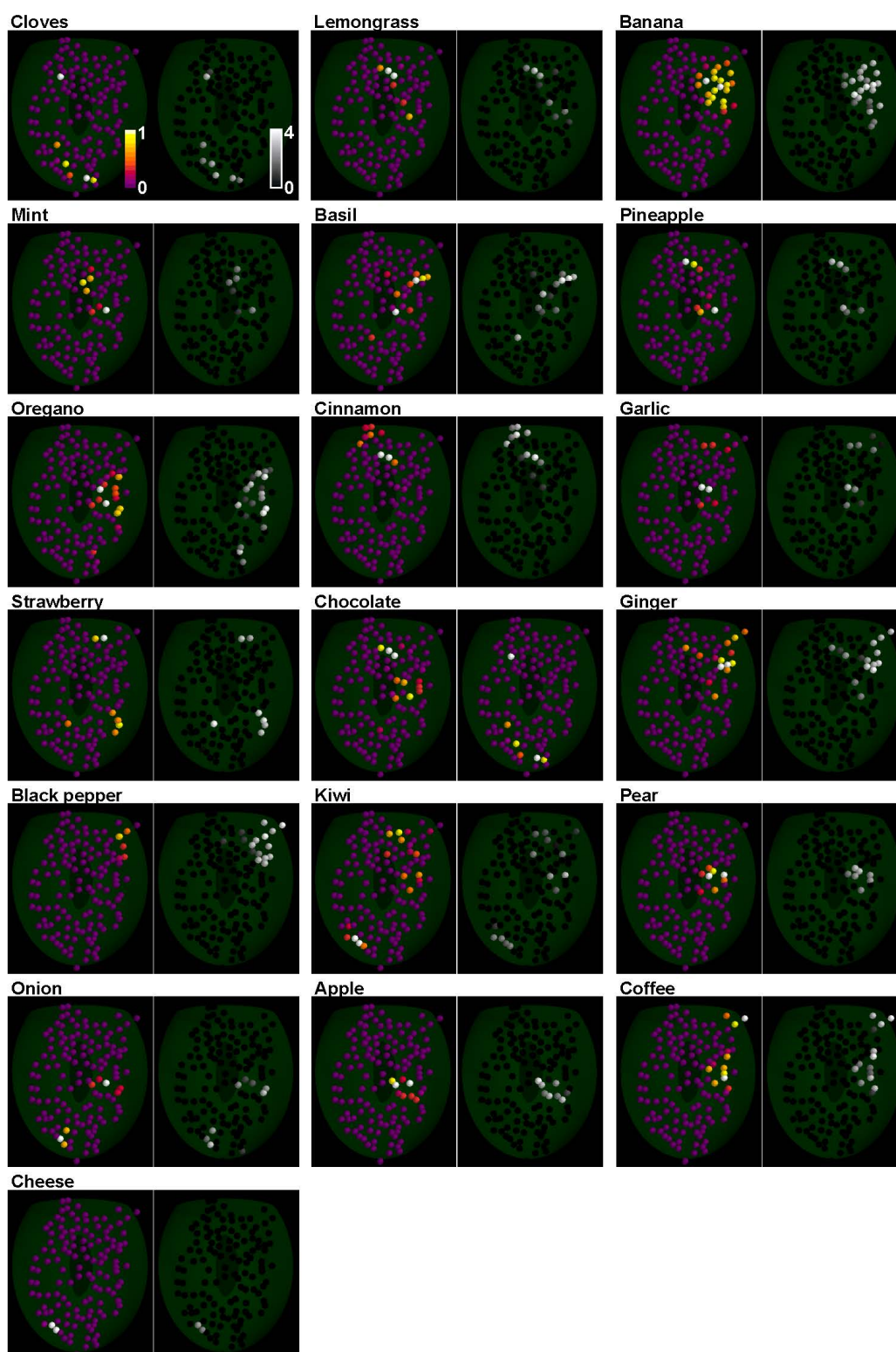

**Figure S3.** Input (left) and average activity (right) for all odors, color coded as in Fig.6 and 7.

Supplement: Supplementary file 3 [file Image3.PDF]
