# Supplementary material for: Glomerular and Mitral-Granule Cell Microcircuits Coordinate Temporal and Spatial Information Processing in the Olfactory Bulb
Source: Front Comput Neurosci. 2016 Jul 14;10:67. doi: 10.3389/fncom.2016.00067 (PMC4943958; doi:10.3389/fncom.2016.00067)
Supplement: Supplementary file 4 [file Image4.PDF]

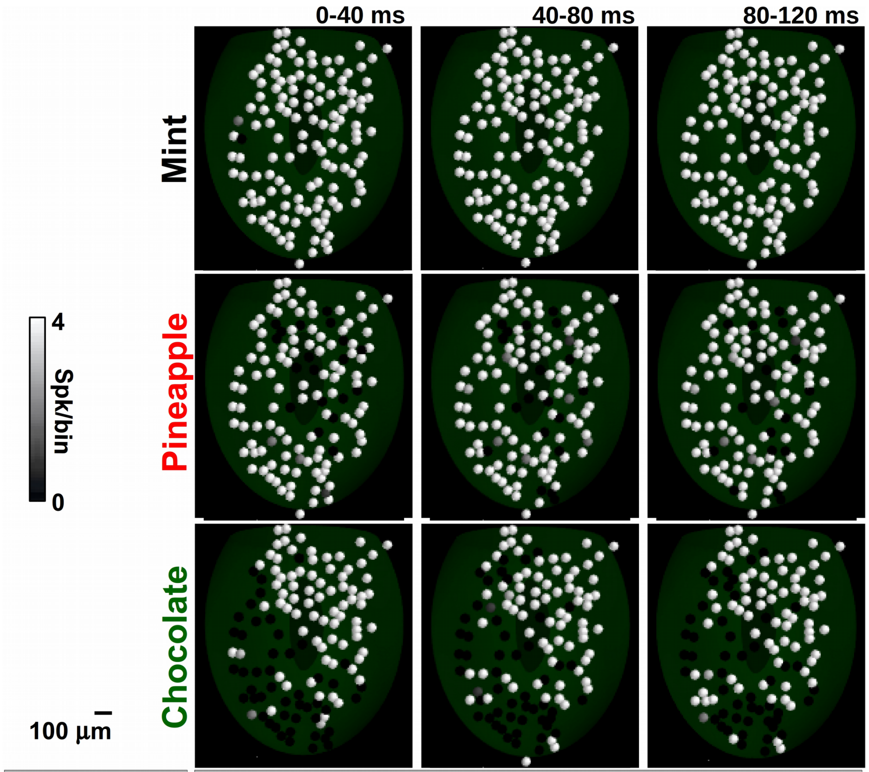

Odors	overlap		
Mint, Pine.	0.793 ± 0.037	0.913 ± 0.012	0.901 ± 0.007
Mint, Choc.	0.731 ± 0.037	0.891 ± 0.019	0.898 ± 0.015
Pine., Choc.	0.854 ± 0.028	0.948 ± 0.012	0.929 ± 0.011

Figure S4. GCL alone cannot decorrelate mitral cell activity over time
The average activity for mint (top), pineapple (medium) and chocolate (bottom) at different time bins during a sniff after learning mint, kiwi, and cloves. Note that, under this condition, odor learning leads to the formation of a diffuse cloud of potentiated GC synapses (see Figs. S3-S4). The spatial overlap for each pair is shown in the table at the bottom for each time window, and shows no decorrelation over time.
